# Ten simple rules for executing an inherited research plan in computational biology

**DOI:** 10.1371/journal.pcbi.1014357

**Published:** 2026-06-12

**Authors:** Sahar Javaheri Tehrani, Toni Ingolf Gossmann

**Affiliations:** Department of Computational Systems Biology, Faculty of Biochemical and Chemical Engineering, TU Dortmund University, Dortmund, Germany; Carnegie Mellon University, UNITED STATES OF AMERICA

## Abstract

Trainees in computational biology frequently inherit research plans whose aims, datasets, analytical strategies, and technical constraints were defined before their arrival. These plans often emerge from grants, collaborations, legacy codebases, shared high-performance computing environments, or partially completed analyses. While such plans provide a useful scaffold, they rarely specify all implementation details, prior assumptions, evaluation criteria, or dependencies needed for reliable execution. The transition from inheriting a partially articulated plan to producing reproducible results therefore creates an *execution gap*: a phase in which trainees must reconstruct what the project is, which elements are fixed, which remain negotiable, and which technical or organizational assumptions need to be tested before full-scale analysis begins. In this Ten Simple Rules article, we provide a practice-oriented framework for stabilizing inherited computational biology projects before workflows, benchmarks, and decision paths become entrenched. We do not claim that the individual practices described here are novel in isolation. Rather, our contribution is to organize familiar practices into a sequenced framework for a recurrent but under-articulated phase of computational research: inherited-plan execution. Computational biology makes this phase especially important because projects often combine heterogeneous datasets, fragile software environments, undocumented preprocessing choices, benchmarking assumptions, distributed collaborators, and asymmetrical access to contextual knowledge. By making this transition visible and operational, the rules aim to help trainees, supervisors, and collaborators reduce ambiguity, test feasibility, document decisions, and support reproducible and equitable project execution under real-world constraints.

## Introduction

**Fig 1:**
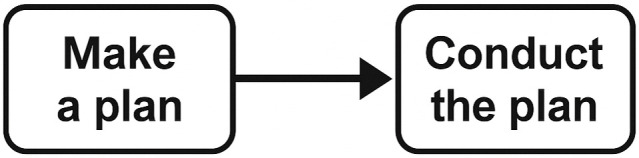
Overview of inherited-plan execution. Research is often described as a linear sequence: plan definition → plan execution. In practice, an intermediary “execution gap” arises where missing context, unclear responsibilities, undocumented assumptions, or untested technical dependencies create friction. The ten rules in this article provide structure for navigating this gap before reproducible analysis begins.

Computational biology research is often described as a simple sequence: define a research plan, then execute it (Fig 1). In practice, however, many trainees do not design projects from scratch. They join ongoing work shaped by grant proposals, collaborations, laboratory agendas, prior analyses, inherited code, or shared computational infrastructure. These inherited projects typically include predefined questions, datasets, and methodological directions, but often lack the operational detail required for reliable computational execution.

A useful distinction is between inheriting a *research idea* and inheriting a *research plan*. An inherited idea leaves methodological design largely open. By contrast, an inherited plan embeds prior commitments such as selected datasets, analytical frameworks, benchmark choices, software environments, collaboration expectations, and timelines, while leaving many implementation details unspecified. This article focuses on the latter case, where trainees must translate partially specified plans into executable workflows.

This situation creates a key asymmetry: core conceptual and technical decisions are often made before the person responsible for execution joins the project. As a result, trainees must reconstruct not only technical steps but also prior assumptions about feasibility, data state, preprocessing choices, evaluation criteria, and acceptable deviations from the original plan. In computational biology, this reconstruction is rarely a purely intellectual exercise. It is distributed across code repositories, undocumented scripts, workflow managers, container files, cluster settings, prior figures, email threads, shared drives, collaborator expectations, and tacit supervisory knowledge. We refer to this reconstructive phase as the *execution gap*.

The execution gap is not resolved by standard onboarding processes [1], which typically assume that project structure, workflows, and expectations are already stable. In inherited computational projects, however, these elements are often only partially specified. Before standard guidance on reproducible workflows, version control, benchmarking, or reporting can be applied, the trainee often has to determine what the workflow is, why specific choices were made, which parts of the plan remain scientifically justified, and which constraints are historical rather than necessary.

This distinction is important for computational biology because the field is inherently hybrid and collaborative. Computational biology projects may be embedded in experimental laboratories, clinical consortia, chemical biology collaborations, bioinformatics cores, or multi-institutional data-science teams. As a result, project ownership, methodological authority, and data access are often distributed across people with different expertise and priorities. The same project may require biological interpretation, statistical modeling, software engineering, data stewardship, high-performance computing, and domain-specific knowledge. These overlapping responsibilities make inherited plans especially vulnerable to ambiguity, because no single person may hold the full context.

The purpose of the rules presented here is therefore not only to guide execution, but to help create enough structure to support deliberate decisions when the project is not yet fully specified. In computational settings, analyses can be generated rapidly and at low cost, allowing substantial apparent progress even when underlying assumptions remain unresolved. A model can be trained, a figure can be produced, or a benchmark can be run long before the dataset, evaluation criteria, or preprocessing decisions have been stabilized. This makes the execution gap particularly consequential: unresolved ambiguity may accumulate silently until it affects interpretation, reproducibility, authorship expectations, or project feasibility.

Drawing on supervisory experience across more than 30 computational projects, we present ten practical rules for stabilizing inherited-plan execution. This experience spans projects in population genomics, transcriptomics, computational systems biology, benchmarking, and collaborative data science. The manuscript is practice-based rather than a formal empirical evaluation. We therefore present the rules as operational guidance derived from recurring supervisory patterns, not as a validated intervention. Their goal is to make a commonly experienced but weakly articulated phase of computational research visible, discussable, and easier to navigate.

### Relation to prior work

This article is grounded in supervisory experience rather than formal educational theory, but the challenges we describe align with established work on research training and mentoring. Studies of the hidden curriculum and cognitive apprenticeship show that expectations about independence, communication, and execution are often implicit, and that expert reasoning must be made visible during skill development [2–5]. In computational research, initiatives such as Software Carpentry and prior Ten Simple Rules articles emphasize structured onboarding, reproducibility, version control, workflow design, and collaboration [6–8].

Our contribution is complementary to these literatures. Prior Ten Simple Rules articles on reproducible computational research, Git and GitHub, and funded project execution provide essential guidance once a project has entered a relatively stable execution state [7–9]. However, inherited computational projects often present an earlier problem: the trainee must first reconstruct what the project actually is before reproducibility, version control, or project-management practices can be applied effectively. The hidden curriculum and cognitive apprenticeship literatures explain why tacit expectations matter, but they do not provide a computational biology-specific operational sequence for reconstructing inherited plans that are distributed across datasets, code, infrastructure, collaborators, and incomplete documentation.

The execution gap also overlaps with broader interpretive challenges in computational and evolutionary biology. As discussed previously [10], computational projects may generate substantial analytical output before a stable narrative or interpretive framework has been established. In inherited projects, this problem is amplified because trainees often inherit partially developed analyses without full access to the reasoning that originally motivated them. Early stabilization is therefore not only a technical process, but also an interpretive one: clarifying which questions the project is actually positioned to answer and which analytical directions are exploratory rather than confirmatory.

We therefore do not claim novelty at the level of individual practices. Clarifying expectations, documenting decisions, defining milestones, and communicating regularly are established practices. The novelty of the present article lies in formalizing how these practices depend on one another during inherited computational project execution. For example, failures and uncertainty must first be surfaced before they can be documented meaningfully; feasibility must be tested before stable milestones can be defined; and stable execution cannot be declared before evaluation criteria, datasets, and preprocessing assumptions have been clarified and fixed. During this transitional phase, familiar practices serve a different functional role. They are not merely tools for efficient execution; they are mechanisms for reconstructing the plan, redistributing contextual knowledge, testing hidden assumptions, and making decision authority explicit.

This framing also clarifies why broad applicability does not make the article discipline-agnostic. Computational biology overlaps with bioinformatics, systems biology, experimental biology, medical genomics, chemical biology, and collaborative data science. Its projects are often embedded in other disciplines through collaborations, shared samples, shared infrastructure, and domain-specific interpretation. The execution gap is therefore central to computational biology precisely because the field frequently operates at these interfaces. Practices described here may transfer to other data-intensive fields, but the combination of heterogeneous biological data, rapidly evolving software, benchmarking norms, infrastructure dependence, and distributed collaborators makes the problem especially acute in computational biology.

#### Common execution pitfalls.

Inherited computational projects frequently destabilize through recurring patterns: (i) implicit or underspecified plans, (ii) unrealistic feasibility assumptions, (iii) silent methodological pivots, (iv) perfectionistic over-execution, (v) weak contextual alignment, (vi) infrastructure-related fragility, and (vii) unclear role expectations within the project. In computational biology, these pitfalls often take concrete forms: undocumented preprocessing choices, changed genome annotations, inconsistent metadata, hidden normalization assumptions, dependency conflicts, stochastic workflow outputs, data leakage in benchmarking, cluster-specific resource assumptions, or collaborator expectations that were never translated into executable criteria.

The ten rules map onto these failure modes and aim to make both technical and organizational dimensions of execution explicit. The rules are sequenced from recognizing the execution gap, to reconstructing the inherited plan, to testing feasibility, documenting decisions, managing adaptation, and finally declaring the transition to stable execution.

## Rule 1: Recognize that inherited projects are not immediately executable

Trainees often enter a project expecting to begin implementation, as if a defined research plan already exists. In inherited computational projects, however, what is provided is rarely an execution-ready plan. It is more often a collection of partially specified elements: datasets, previous analyses, code fragments, figures, grant aims, collaborator expectations, infrastructure assumptions, and informal supervisory explanations.

This creates an execution gap between what appears to be a defined project and what can actually be implemented. Without recognizing this gap, trainees may proceed directly to coding or analysis, leading to confusion, hidden dependencies, or misaligned effort. In computational biology, this can produce misleading apparent progress: a pipeline may run, but on the wrong data version; a model may train, but with leaked labels; a figure may be produced, but under preprocessing assumptions no collaborator has agreed upon [11].

The first step is therefore not to start implementing, but to assess what is already specified, what is assumed, and what remains unclear. Recognizing this gap repositions the task from executing a plan to interpreting and stabilizing it before further work proceeds.

**Example.** A trainee begins implementing a predictive model based on a project description, only to discover that the cohort definition, preprocessing steps, train-test split, and evaluation metric were never fixed. Rather than continuing with ad hoc choices, they recognize that the project is not yet execution-ready and shift focus to reconstructing the required components.

## Rule 2: Anchor the plan in scientific purpose and expected outcomes

Once it is recognized that the project is not yet execution-ready (Rule 1), the next step is to clarify its scientific purpose and expected outcomes. This includes understanding why the project matters, what question it aims to answer, and what would count as a useful result. Without this anchor, trainees may optimize technical details that do not advance the project’s scientific purpose.

Engagement with the literature is important at this stage, but it should be structured rather than exhaustive. Supervisors should provide key references and context to help situate the project, while trainees progressively build understanding as the project develops. The goal is not complete domain mastery upfront, but agreement about the intended contribution, evaluation criteria, and acceptable trade-offs.

In inherited computational projects, scientific purpose is also needed to interpret technical constraints. For example, whether a missing metadata field is fatal, whether a benchmark is appropriate, or whether an alternative dataset changes the project’s scope depends on the scientific question being asked.

**Example.** A trainee inherits a single-cell analysis project and initially focuses on optimizing clustering parameters. A short discussion of the scientific aim reveals that the project is not primarily about clustering quality, but about identifying reproducible cell-state differences across treatment conditions. This changes which quality-control steps, comparisons, and validation criteria matter most.

## Rule 3: Identify stakeholders, roles, and sources of authority

In inherited projects, decisions about scope, methods, and priorities are distributed across multiple actors, including supervisors, collaborators, prior contributors, core facilities, data providers, and technical support staff. Understanding who shapes the plan and who controls key resources is essential for navigating the execution gap.

Role ambiguity is a common source of instability. Trainees may oscillate between service-oriented tasks, such as maintaining pipelines or producing figures, and narrative-driving work, such as shaping hypotheses or evaluation criteria, without clarity about what is expected or valued. As a result, effort may accumulate without advancing the project’s core scientific direction.

Power asymmetries can make clarification difficult. Trainees may hesitate to question inherited decisions, request missing documentation, or renegotiate timelines, especially when supervisors are busy, collaborators are senior, language barriers exist, or employment conditions are precarious. The framework proposed here is not intended to assume ideal supervisory conditions. A written summary of who controls data access, who can modify the pipeline, which benchmark definitions are fixed, and which methodological decisions require approval gives trainees a stable reference point that does not depend entirely on repeated informal negotiation.

When direct questioning is difficult, trainees can use indirect clarification strategies: framing questions in terms of avoiding wasted effort, preparing short written summaries for confirmation, documenting assumptions before acting on them, asking trusted peers or senior researchers to review the project state, or using meeting notes to create a non-confrontational paper trail. Supervisors and senior collaborators have a corresponding responsibility to make sources of authority and constraint explicit rather than expecting trainees to infer them.

**Example.** A trainee inherits a workflow maintained by a previous student but used across several collaborations. Clarifying that a senior postdoc manages the software environment, that one collaborator controls data access, and that another depends on specific output formats prevents unintended changes and reveals which parts of the workflow can be modified safely.

## Rule 4: Reconstruct the inherited plan from its elements

Once the scientific purpose (Rule 2) and sources of authority (Rule 3) are clarified, the project’s concrete elements can be assembled into a visible structure. This is a reconstruction task, not yet an execution task.

Identify and organize the components that define the project in practice. These typically include datasets, metadata, preprocessing steps, analytical methods, benchmark choices, evaluation criteria, prior results, code repositories, container or environment files, workflow managers, access permissions, and computational infrastructure [12]. The aim is to make the implicit plan explicit enough to understand how its parts fit together and which missing links must be resolved.

Inherited projects lie on a spectrum from relatively well-defined scaffolds to loosely specified directions. Assessing where a project falls on this spectrum helps determine the size of the execution gap and whether reconstruction primarily involves consolidating existing elements or actively co-developing missing ones with the supervisor.

**Example.** A method-comparison project consists of a draft figure, a dataset used in earlier analyses, and a partially implemented pipeline. Bringing these elements together reveals that the benchmark dataset was filtered differently across analyses, evaluation criteria were never fixed, and the workflow depends on an undocumented local software environment. The trainee pauses full execution and reconstructs the plan before producing further results.

## Rule 5: Write down the visible working plan

After clarifying the inherited scaffold, turn it into a shared working plan. This plan should make visible what the project is now understood to be, what constraints it carries, and what remains open to adaptation.

A useful working plan includes: (i) the research question; (ii) the intended contribution; (iii) fixed versus flexible elements; (iv) expected outputs; (v) known risks; and (vi) immediate next steps. The point is not bureaucratic completeness, but to create a shared operational document that makes the project executable rather than assumed [2,3,13]. A short teach-back summary by the trainee is often enough to reveal remaining mismatches.

In inherited computational projects, the visible working plan should also name technical assumptions explicitly: dataset versions, reference genomes, preprocessing choices, software environments, benchmark definitions, and criteria for deciding whether a pilot has succeeded or failed. This shifts tacit knowledge into a form that can be reviewed, corrected, and handed forward.

**Example.** A trainee summarizes an inherited population-genomics project in one page, including the reference genome version, sample inclusion criteria, planned comparison, benchmark choice, filtering assumptions, and unresolved questions about missing metadata. The supervisor corrects two key misunderstandings before substantial computation begins.

## Rule 6: Use exploratory pilots to define feasible milestones

Once a visible plan exists, initial execution should remain exploratory. Rather than immediately committing to full-scale analysis, use small pilots to probe feasibility and translate emerging insights into bounded milestones.

Exploratory pilots test whether planned steps can be executed under real data and computational conditions [6,9,14,15]. They often reveal hidden dependencies, unstable assumptions, resource constraints, stochastic variation, or mismatches between intended and actual workflows [16]. These findings should not remain isolated observations, but should be used to define or revise milestones that reflect what is realistically achievable.

Milestones should therefore emerge from exploratory work and specify entry conditions, exit criteria, and a realistic time frame. This is especially important when full-scale analyses are expensive, when workflows run on shared clusters, or when early outputs may be mistaken for final results. A pilot is not a failed execution; it is a structured test of whether execution is ready to begin.

**Example.** A trainee begins with a small pilot on 10 representative samples rather than launching an RNA-seq analysis across 200 samples. The pilot reveals missing metadata, inconsistent genome annotation files, and excessive memory use on the available cluster partition. Instead of continuing with ad hoc adjustments, the trainee defines a milestone to resolve data completeness, annotation consistency, and resource requirements before scaling up.

Rules 7–9 address closely related aspects of early project stabilization. They are separated because they address different problems. Rule 7 concerns the epistemic status of failure and uncertainty; Rule 8 concerns how information is communicated and preserved; Rule 9 concerns when accumulated changes amount to re-planning rather than refinement. In practice, these steps operate iteratively.

## Rule 7: Treat failure, debugging, and uncertainty as information

During early exploratory phases, trainees may hesitate to report failures or uncertainty, particularly when expectations are unclear. It is therefore essential to normalize early reporting. In inherited computational projects, failures often reflect hidden assumptions in prior code, incomplete documentation, fragile infrastructure, or changed data conditions rather than lack of competence [17].

Report failed runs, unexpected results, unstable benchmarks, and unresolved uncertainty as soon as they arise. A failed pilot or broken workflow is not just an obstacle; it is evidence about the actual state of the inherited scaffold. Surfacing this information early reduces the risk of silent workarounds and reveals dependencies that would otherwise remain hidden.

This rule is distinct from documentation. Before a decision can be documented, the uncertainty has to be treated as legitimate information rather than as a personal failure to be hidden. Making instability visible is therefore a prerequisite for understanding the system as it actually operates.

**Example.** A workflow repeatedly crashes on a high-performance computing cluster. Rather than treating this as personal error, the trainee reports it immediately. The problem turns out to be an inherited resource assumption: the original pipeline was developed on a larger-memory node that is no longer available under the current allocation.

## Rule 8: Make communication and documentation shared and persistent

As instability and uncertainty are surfaced, they must be made visible to others and preserved over time. Inherited projects drift when decisions are made but not communicated, or communicated but not recorded. Communication and documentation therefore serve complementary roles: discussion aligns understanding, and documentation preserves its rationale [7,8,17,18].

Use regular, structured communication to review what was implemented, what was learned, what remains blocked, and what decisions are required. Record key decisions, parameter choices, dataset versions, software environments, abandoned approaches, and changes in evaluation criteria in a lightweight but persistent form. This keeps the working plan current and makes the project intelligible to collaborators and future contributors.

It may be tempting to treat exploratory analyses or intermediate results as too preliminary to document. In practice, these stages are often the most important to capture. Unlike final publications, which typically report selected outcomes, project execution depends on preserving alternative paths, failed attempts, and evolving assumptions so that decisions remain interpretable.

Documentation also helps mitigate power asymmetries. A written summary of assumptions, blockers, and proposed next steps allows trainees to request clarification without direct confrontation and gives supervisors a concrete opportunity to correct misunderstandings. The goal is not exhaustive record-keeping, but maintaining a shared and traceable understanding of the evolving plan.

**Example.** A trainee switches to a different normalization strategy after encountering unexpected batch effects. Because the change, rationale, affected datasets, and consequences for earlier figures are recorded, later comparisons remain interpretable and collaborators can assess whether the change alters the scope of the project.

## Rule 9: Treat re-planning as deliberate adaptation, not drift

As uncertainty becomes visible (Rule 7) and decisions are made explicit (Rule 8), it often becomes clear that the current plan requires adjustment. Re-planning is therefore a normal outcome of stabilizing an inherited project rather than a sign of failure [6,15].

Not all changes are equal. Minor adjustments refine the project without affecting its direction, whereas major changes, such as replacing datasets, redefining evaluation criteria, changing the target biological question, or altering the methodological framework, can affect feasibility, timelines, authorship, and scientific scope. These should be treated as explicit decisions rather than incremental modifications.

This rule is distinct from communication and documentation. A project may document many changes yet still fail to recognize that the accumulated changes have redirected the project. Distinguishing between refinement and redirection reduces gradual drift and supports a transition to stable execution.

**Example.** Initial analyses suggest that the available whole-genome sequencing dataset lacks sufficient coverage depth for reliable structural variant detection. Rather than continuing with incremental parameter adjustments, the trainee raises this as a project-level issue. After discussion, the plan is revised to focus on small-variant benchmarking using a different evaluation framework and adjusted success criteria. Treating this as explicit re-planning clarifies expectations and prevents silent divergence from the original project scope.

## Rule 10: Explicitly declare the transition to stable execution

The preceding steps (Rules 1–9) focus on interpreting, reconstructing, and stabilizing an inherited research plan. However, this process is only complete once the project has reached a state where it can be executed under fixed conditions.

At this point, it is essential to explicitly declare that the plan has entered a stable execution phase. This includes fixing datasets, preprocessing decisions, evaluation metrics, benchmark definitions, software environments, and major comparison criteria. From this stage onward, results should be generated and interpreted without further modification of the analytical framework [15,19,20].

Making this transition explicit serves two purposes. First, it reduces the risk that exploratory adjustments influence final conclusions. Second, it marks the resolution of the execution gap: the inherited plan has been made visible, tested, and stabilized sufficiently to support reproducible and interpretable results. In collaborative, consortium, or benchmarking settings, this transition may also justify preregistering core analysis decisions or evaluation criteria so that confirmatory results are generated under explicitly fixed conditions [20].

Subsequent changes should be treated as re-planning rather than incremental adjustment. Minor refinements may still be appropriate, but major changes—such as altering datasets, redefining evaluation criteria, replacing core methods, or changing validation strategy—should be explicitly discussed, as they may affect the validity, scope, or deliverables of the project.

**Example.** After running pilots and refining a workflow, a trainee finalizes preprocessing steps, benchmark datasets, software versions, and evaluation metrics. These choices are documented and no longer modified during benchmarking. A later proposal to switch datasets is treated as a major re-planning decision rather than a routine adjustment.

## Summary table of the ten rules

Table 1 provides a concise overview of all ten rules and can be used for onboarding, lab mentoring, or teaching modules.

**Table 1:** Overview of the reconstruction, stabilization, and execution of inherited research plans in computational biology.

Rule	Core Question	Key Take-away
1	Is this project ready for execution?	Recognize that inherited projects are not immediately executable but consist of fragmented elements requiring interpretation.
2	Why does this project exist and what counts as success?	Clarify scientific purpose, expected outputs, and success criteria.
3	Who shapes the plan and who decides what?	Identify stakeholders, roles, authority, and sources of constraint, including power asymmetries.
4	What are the concrete elements of the project?	Reconstruct datasets, code, prior analyses, infrastructure, and expectations into a coherent structure.
5	What is the working plan?	Make the project explicit as a shared, written plan with goals, constraints, assumptions, and risks.
6	What is feasible in practice?	Use exploratory pilots to test assumptions and define realistic milestones.
7	What do failures and instability reveal?	Treat debugging, negative results, and uncertainty as information about the inherited scaffold.
8	Are changes visible and traceable?	Use structured communication and documentation to preserve decisions, assumptions, and rationale.
9	Are we refining or redirecting the plan?	Treat re-planning as deliberate adaptation and distinguish minor refinements from major changes.
10	When is the project ready for stable execution?	Explicitly fix evaluation criteria and declare the transition from development to execution.

## Future perspectives

As computational biology projects grow in scale and complexity, inherited plans will increasingly involve multi-institutional collaborations, heterogeneous datasets, containerized workflows, shared codebases, and high-performance computing infrastructure. The interpretive demands placed on trainees are therefore likely to increase rather than diminish.

One practical extension of the concepts presented here is to move elements of the execution gap earlier in the research lifecycle. In structured PhD recruitment or graduate training programs, aspects of inherited-plan reconstruction could be made partially visible during onboarding or selection. For example, applicants or new trainees could be provided with representative materials—such as a draft figure, dataset description, prior pipeline, or incomplete workflow—and asked to articulate how they would interpret the project, identify uncertainties, or define initial next steps. The goal would not be to resolve the project in advance, but to expose the kinds of reasoning required during early project stabilization.

Similarly, graduate schools and research groups could incorporate lightweight onboarding modules that explicitly address inherited-plan execution. Such modules could include identifying fixed versus flexible elements, evaluating feasibility through small pilots, distinguishing exploratory from confirmatory phases, documenting assumptions, and recognizing when technical instability indicates a project-level problem rather than an individual mistake.

The practical motivation for these rules emerged repeatedly across inherited computational projects. For example, during the development of a population-genomics benchmarking workflow in our prior work [21], exploratory analyses revealed inconsistent preprocessing assumptions, undocumented filtering decisions, and evolving benchmark criteria across collaborators. Although the workflow initially appeared technically functional, stabilizing the project required reconstructing dataset provenance, clarifying evaluation criteria, and explicitly separating exploratory analyses from confirmatory comparisons before reproducible benchmarking could proceed. Resolving these inconsistencies required substantial re-planning, temporarily stalled progress, and created friction among collaborators regarding which analyses were considered methodologically comparable. Similar patterns recurred across multiple projects and motivated the present framework.

More broadly, systematically studying the execution gap as a distinct phase of computational research represents a promising direction for future work. Potential outcome measures could include time-to-first-reproducible-result, stability of pipelines across personnel transitions, frequency of major re-planning events, number of undocumented methodological changes detected during handover, or trainee confidence in identifying decision authority. Such investigations would complement existing work on computational reproducibility and mentoring by focusing on an earlier and currently under-structured stage of the research process.

## Conclusion

Executing an inherited research plan is one of the most common yet least articulated challenges in computational biology training. The gap between high-level planning and day-to-day execution is where projects can lose alignment, feasibility, and momentum. When commitments, roles, preprocessing assumptions, software dependencies, and evaluation criteria remain implicit, even well-intended plans can drift or stall. In computational settings in particular, apparent progress can be made despite unresolved misalignment, because analyses can be generated rapidly without stabilizing underlying assumptions.

These ten simple rules treat inherited-plan execution as a structured process of reconstructing, stabilizing, and ultimately executing a partially specified research plan. They provide practical mechanisms for clarifying conceptual alignment, defining bounded milestones, stress-testing feasibility, separating exploration from confirmation, documenting interpretive decisions, and managing deliberate re-planning. The individual practices are familiar, but their interdependence during inherited computational project execution is what makes the execution gap a distinct and useful framing.

These recommendations do not assume that all trainees work in supportive or fully communicative environments. Power asymmetries, limited supervisory availability, language barriers, precarious employment, and resource constraints can restrict a trainee’s ability to question inherited decisions or renegotiate plans [22]. In such contexts, written summaries, persistent documentation, peer consultation, and explicit decision points may provide partial mechanisms for reducing ambiguity, though they cannot remove structural constraints. Acknowledging these realities underscores the importance of transparent mentoring cultures and institutional support structures alongside individual execution practices.

Making the execution gap visible reframes inherited plans from fixed instructions into objects that must be actively interpreted, tested, negotiated, and stabilized before reliable computational execution can begin.
